# Assessment of Liver Stiffness Regression and Hepatocellular Carcinoma Risk in Chronic Hepatitis C Patients after Treatment with Direct-Acting Antiviral Drugs

**DOI:** 10.3390/medicina57030210

**Published:** 2021-02-26

**Authors:** Martynas Ridziauskas, Birutė Zablockienė, Ligita Jančorienė, Artūras Samuilis, Rolandas Zablockis, Aušrinė Jackevičiūtė

**Affiliations:** 1 Vilnius University Faculty of Medicine, M.K. Ciurlionio 21, LT-03101 Vilnius, Lithuania; ausrine.jackeviciute@mf.stud.vu.lt; 2 Center of Infectious Diseases, Vilnius University Hospital Santaros Klinikos, LT-08406 Vilnius, Lithuania; birute.zablockiene@santa.lt (B.Z.); ligita.jancoriene@santa.lt (L.J.); 3 Clinic of Infectious Diseases and Dermatovenerology, Institute of Clinical Medicine, Vilnius University Faculty of Medicine, M.K. Ciurlionio 21, LT-03101 Vilnius, Lithuania; 4 Center of Radiology and Nuclear Medicine, Vilnius University Hospital Santaros Klinikos, LT-08661 Vilnius, Lithuania; arturas.samuilis@santa.lt; 5 Department of Radiology, Nuclear Medicine and Medical Physics, Faculty of Medicine, Vilnius University, M.K. Ciurlionio 21, LT-03101 Vilnius, Lithuania; 6 Center of Pulmonology and Allergology, Vilnius University Hospital Santaros Klinikos, Santariskiu 2, LT-08661 Vilnius, Lithuania; rolandas.zablockis@santa.lt; 7 Clinic of Chest Diseases, Immunology and Allergology, Faculty of Medicine, Institute of Clinical Medicine, Vilnius University, M.K. Ciurlionio 21, LT-03101 Vilnius, Lithuania

**Keywords:** hepatitis C, liver stiffness, direct-acting antiviral drugs, liver ultrasound elastography, hepatocellular carcinoma

## Abstract

*Background and Objectives*: Chronic hepatitis C virus infection affects about 71 million people worldwide. It is one of the most common chronic liver conditions associated with an increased risk of developing liver cirrhosis and cancer. The aim of this study was to evaluate changes in liver fibrosis and the risk of developing hepatocellular carcinoma after direct-acting antiviral drug therapy, and to assess factors, linked with these outcomes. *Materials and Methods*: 70 chronic hepatitis C patients were evaluated for factors linked to increased risk of *de novo* liver cancer and ≥ 20% decrease of ultrasound transient elastography values 12 weeks after the end of treatment. *Results*: The primary outcome was an improvement of liver stiffness at the end of treatment (*p* = 0.004), except for patients with diabetes mellitus type 2 (*p* = 0.49). Logistic regression analysis revealed factors associated with ≥ 20% decrease of liver stiffness values: lower degree of steatosis in liver tissue biopsy (*p* = 0.053); no history of interferon-based therapy (*p* = 0.045); elevated liver enzymes (*p* = 0.023–0.036); higher baseline liver stiffness value (*p* = 0.045) and absence of splenomegaly (*p* = 0.035). Hepatocellular carcinoma developed in 4 (5.7%) patients, all with high alpha-fetoprotein values (*p* = 0.0043) and hypoechoic liver mass (*p* = 0.0001), three of these patients had diabetes mellitus type 2. *Conclusions*: Liver stiffness decrease was significant as early as 12 weeks after the end of treatment. Patients with diabetes and advanced liver disease are at higher risk of developing non-regressive fibrosis and hepatocellular carcinoma even after successful treatment.

## 1. Introduction

Hepatitis C virus (HCV) affects about 71 million people worldwide. Chronic HCV infection is one of the most common chronic liver diseases, characterized by progressive liver fibrosis and increased risk of developing hepatocellular carcinoma (HCC) [1]. Liver cirrhosis develops in about 10–30% of patients within 20 years of infection [1,2,3]. About 1% to 4% of patients with HCV-induced liver cirrhosis are diagnosed with HCC each year [3]. Previously, patients with chronic HCV infection used to be treated with interferon- based regimens. After reaching sustained virologic response (SVR), a reduction in portal hypertension [4,5] and fibrosis regression [6,7] are usually observed. In 3010 patients with HCV-induced liver cirrhosis successfully treated with interferon, the incidence of hepatic fibrosis was reduced by 49% [8]. However, the relevant data are inconsistent in the case of decompensated cirrhosis [9].

Since 2013 in the world, and since 2015 in Lithuania, a new generation of direct-acting antiviral drugs (DAAs) has been introduced into clinical practice. Thess novel and particularly effective HCV treatments require a shorter duration and causes fewer side effects than interferon-based therapy. DAA treatment leads to SVR in up to 95% of cases [10,11]. The new treatment (contrary to interferon regimens) is also available for patients with decompensated liver cirrhosis [12]. However, data on the impact of this successful next-generation treatment on liver fibrosis regression are still lacking.

Hepatic inflammatory activity and stage of liver fibrosis used to be assessed by histological examination prior to treatment. Liver biopsy was considered the gold standard for fibrosis assessment; however, it is associated with longer hospitalization, higher risk of complications, and increased treatment costs [13]. The accuracy of the histological examination depends on the sampling technique, sample size and experience of the examiner [14]. In addition, liver biopsy is rarely repeated after the treatment due to the invasiveness of the procedure and ethical considerations. Therefore, non-invasive liver fibrosis assessment methods recently were widely introduced into clinical practice. Evaluation of liver stiffness using transient elastography (TE, most known as FibroScan) is one of the most used non-invasive liver fibrosis assessment methods. TE is also convenient for assessing the dynamics of liver stiffness. Studies have shown that an SVR after DAA treatment is linked to a decrease in liver stiffness, especially in patients with advanced HCV-induced liver fibrosis [9,15,16]. There is also a reduction in fibrosis scores FIB-4 (which includes the patient’s age, aspartate aminotransferase (AST), alanine aminotransferase (ALT) levels and platelet count) and APRI (liver fibrosis index, calculated by measuring the ratio of AST to platelet count). However, it is not clear whether these results are associated with a reduction in fibrosis or the inhibition of chronic inflammation and interstitial edema in hepatic tissue [15].

Treatment of chronic HCV infection using interferon-based regimens resulted in SVR in up to 50% of cases [17]. Studies have shown that patients who achieved SVR after this treatment had a reduction in liver fibrosis, lower risk of complications, mortality and lower incidence of HCC compared with patients who have not reached SVR [18,19,20,21]. Higher incidence of HCC has been reported in patient groups with diabetes mellitus and HCV genotype 3 [22,23].

DAAs have only recently been introduced for the treatment of hepatitis C; therefore, doubts remain about the effectiveness of this treatment in preventing the development of HCC [17,24,25]. The incidence of HCC development in successfully treated patients could be expected to decrease [16], but studies on the incidence of HCC after treatment with DAA are not yet numerous, and the results are mixed. Conti and co-authors [26] evaluated DAA treatment data from 285 patients with HCV liver cirrhosis. SVR was achieved in 91% of cases. During 24 weeks posttreatment follow-up, HCC was diagnosed in 9 patients (3.2%; 95% CI 1.45–5.90). The authors concluded that SVR elicited by DAAs does not reduce the risk of HCC development over a short follow-up period. In a meta-analysis of 26 studies summarizing chronic HCV infection treated with DAAs (11.523 patients), the treatment was not associated with a higher rate of HCC development (RR 0.68; 95% CI 0.18–2.55, *p* = 0.55) [27]. In the other two large cohort studies, achieved SVR was associated with a 71–76% lower risk of developing HCC [28,29].

In the DAA era, the population of patients has changed-increasingly more patients with advanced liver disease and decompensated cirrhosis are being treated. Individuals with HCV-induced cirrhosis who were treated with DAAs remain at high risk of developing HCC even after achieving SVR and should be monitored further [17]. This could also explain the higher-than-expected rate of HCC development in previously mentioned studies. However, more detailed studies on HCC incidence in patients treated with DAAs are needed to clarify the real factors associated with possible greater risk.

The aim of this study was to evaluate changes in liver stiffness and risk of developing HCC after direct-acting antiviral therapy and to assess factors linked with these out- comes in patients with advanced liver fibrosis treated with DAAs.

## 2. Materials and Methods

### 2.1. Study Population and Design

**Figure 1:**
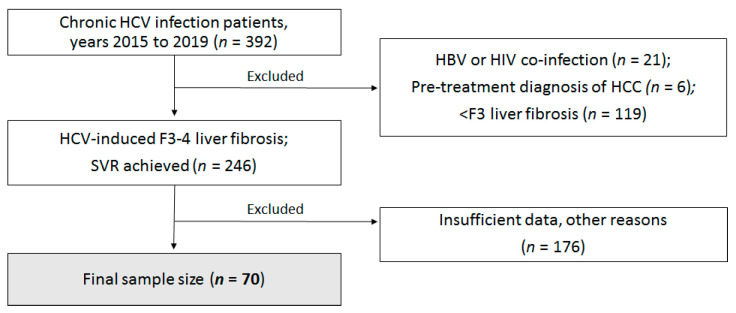
Study flowchart. HCV—hepatitis C virus; HBV—hepatitis B virus; HIV—human immunodeficiency virus; HCC—hepatocellular carcinoma; SVR—sustained virologic response.

This retrospective analytical study was performed in the Center of Infectious Diseases of Vilnius University Hospital Santaros Klinikos (VUH SK). The study population consisted of adult women and men treated in the years 2015 to 2019. All patients had HCV-induced F3 and higher hepatic fibrosis (according to the METAVIR score) or ultrasound transient elastography technique (FibroScan) values greater than 9.5 kPa (in cases when a percutaneous liver biopsy was contraindicated) [30]. Depersonalized health records of all patients who met the inclusion criteria were reviewed. Patients who discontinued DAA treatment, cases with pre-existing HCC diagnosis, patients with HIV or hepatitis caused by other viruses, and cases with insufficient data were excluded from the study. A detailed patient selection process is provided in Figure 1.

From 1 January 2015 to 29 October 2019, 392 patients with chronic HCV were examined and treated in the Center of Infectious Diseases of VUH SK. The retrospective study included 70 subjects who met the mentioned inclusion criteria. The majority of cases were excluded due to insufficient test data or grade F1-2 fibrosis. The study was approved by the Vilnius Regional Biomedical Research Ethics Committee (No.2020/2-1195-681, approved date 13, December 2019).

Patients were evaluated for their comorbidities (obesity, primary arterial hypertension (PAH), diabetes mellitus type 2 (DM2)), possible route and duration of HCV infection, HCV genotype. Patients were assessed by histological examination or FibroScan exam prior to treatment. Histological evaluation of the liver included HAI (histology activity index), degree of hepatic steatosis and fibrosis score according to the METAVIR). The cut-off value for significant steatosis in liver biopsy was ≥30%.

### 2.2. Laboratory Test Data

Baseline and data after 12 weeks of follow-up (SVR12) were evaluated for these factors: HCV viremia, ALT, AST and AFP (alpha-fetoprotein) concentration. Prothrombin index (PTI), GGT (gamma-glutamyltransferase) and platelet count were evaluated before treatment. A significant increase in hepatic biochemical inflammatory parameters was defined as serum ALT or AST > 40 U/L, GGT > 36 U/L. Significant AFP concentration was considered when >5.5 kU/L.

### 2.3. Radiological Data

Baseline, EoT and SVR12 liver tissue stiffness measurements were obtained by ultra- sound transient elastography using M or XL sensors (Echosens FibroScan 402, Paris, France) in left lateral position (with at least 6 h of fasting). The test was considered as correct when the following criteria were met: (1) at least 10 successful measures were obtained; (2) success rate at least 60%; (3) IQR value < 30%. Measures were obtained by intercostal approach on the 4th right intercostal space. Liver stiffness values were categorized as follows: 7.1–8.5 kPa corresponds to F1–F2, 8.6–9.5 kPa-F2, 9.6–12.5 kPa-F3, 12.6–14.5 kPa-F3–F4 and > 14.5 kPa—F4 fibrosis stage according to the METAVIR [30]. Reduction of ≥20% of transient elastography (TE) score was considered significant.

Conventional ultrasound (US) examination of the liver and spleen in grayscale mode with convex probe (Mindray DC-7, C5-2 (3.5 MHz) probe, Shenzhen, China) was performed before and 12 weeks after achieving SVR. Liver size, signs of hepatosteatosis, focal liver lesions and signs of cirrhosis were evaluated. Patients were examined after at least 6 h of fasting. All examinations were performed by the same experienced specialist. Splenomegaly was defined as US spleen length ≥ 20 mm and hepatomegaly as CC (craniocaudal) measure ≥155 mm.

### 2.4. Statistical Analysis

The distribution of continuous variables was evaluated using the Shapiro-Wilk test, histograms and boxplots. Normally distributed continuous variables are expressed as mean (±standard deviation), non-normal distribution data are expressed as median and IQR (Q1–Q3). Data from different groups were compared using Student’s t-test (for normal distribution) or Mann-Whitney-Wilcoxon test (for non-normal distribution). Data were compared by one-way analysis of variance (ANOVA) with Bonferroni correction. Categorical variables were compared with chi-square or Fisher’s exact test. A binary logistic regression model was applied to assess the risk factors. Regression results are expressed as odds ratios (OR) with 95% confidence intervals (CI). Statistical significance was defined when *p* < 0.05. Data were analyzed using the Statistical Package for Social Science software for Windows v21.0 (IBM Corp., Armonk, NY, USA).

## 3. Results

### 3.1. Study Population

The average age of patients was 56.3 (44.8–67.8) years. Sixty percent of patients were males. The body mass index (BMI) median was 27 (25–32) kg/m^2^. Of 57 patients whose BMI was evaluated, 23 were diagnosed with obesity (BMI ≥ 30 kg/m2).

The most common HCV genotypes were 1b (54.3%) and 3 (25.7%). The most common transmission routes were through blood donation or transfusion prior to donor screening introduction in 1993 (54.8%) and intravenous drug abuse (21.4%). Nineteen (27.1%) patients were previously treated with the combination of interferon and ribavirin, and 51 (72.9%) were treatment-naive. DAA treatment regimens are available in Table 1.

Hepatic fibrosis score F4 according to the METAVIR was confirmed histologically in 62 (88.6%) patients. Fibrosis was assessed using TE (instead of histologic examination) in 8 patients.

When comparing F3 to F4 fibrosis score patients. Genotype 3 was found to be statistically significantly more often associated with grade F4 fibrosis than other genotypes (*p* = 0.033). There was no statistically significant distribution of age, gender, obesity, PAH and DM2 when comparing these two groups. More detailed patient demographic and clinical data are available in Table A1.

### 3.2. DAA Treatment Outcomes

#### 3.2.1. Laboratory Test Data

**Figure 2:**
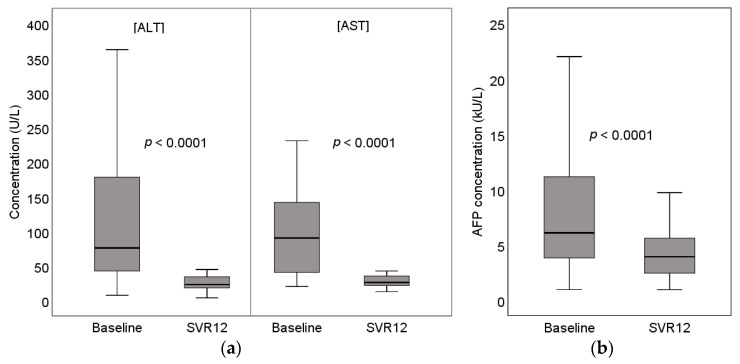
(**a**) Baseline and SVR12 (sustained virologic response after 12 weeks of follow-up) concentration of serum ALT (alanine aminotransferase) and AST (aspartate aminotransferase); (**b**) baseline and SVR12 concentration of serum AFP (alpha-fetoprotein).

All patients enrolled in the study achieved SVR after treatment. There was a statistically significant decrease in all evaluated biochemical markers of hepatitis (ALT, AST, GGT) and AFP (*p* < 0.0001). More information is provided in Table 2 and Figure 2:

#### 3.2.2. Transient Elastography Results

**Figure 3:**
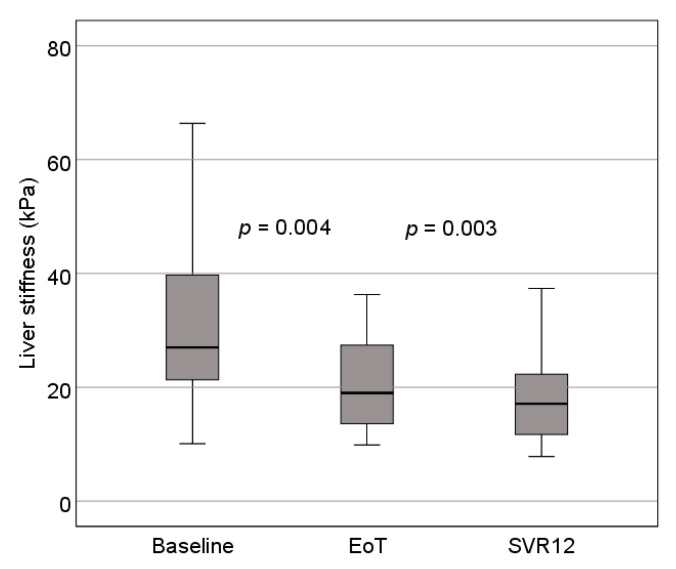
Changes in liver elastography values. EoT—end of treatment; SVR12—sustained virologic response after 12 weeks of follow-up.

Liver stiffness was evaluated before, after and 12 weeks after treatment in 64 of the 70 patients (6 patients had incomplete liver stiffness evaluation). The median values of TE of the liver decreased significantly after treatment (*p* = 0.004). A statistically significant decrease in TE values was found when comparing all data groups: baseline and EoT—*p* = 0.004; baseline and SVR12—*p* = 0.00286; EoT and SVR12—*p* = 0.00470. Detailed information is available in Table 3 and Figure 3.

Assessing the influence of comorbidities, the median decrease of TE values was found insignificant only in the DM2 (*p* = 0.49) group. However, there was no evident in- fluence of obesity or PAH on TE value changes (*p* < 0.05).

#### 3.2.3. Ultrasound Examination Findings

**Figure 4:**
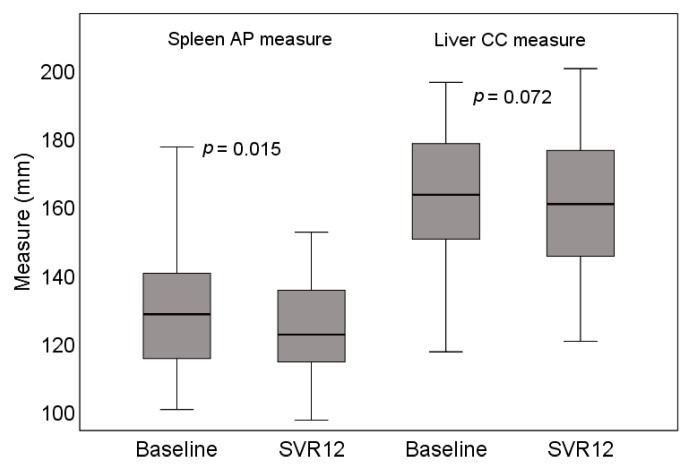
Changes in liver and spleen US measures. AP—anteroposterior; CC—craniocaudal. SVR12—sustained virologic response after 12 weeks of follow-up.

Fifty-seven of 70 patients had a full US assessment (liver, spleen, lymphadenopathy, ascites, and portosystemic collaterals) before treatment and at SVR12. The most common findings were irregular liver contours (71.9%) and irregular texture (61.4%). Hepatomegaly was found in 50.0% and splenomegaly in 28.6% of patients. A statistically significant reduction of spleen (but not liver) size was observed at SVR12 (*p* = 0.015). More detailed information is provided in Table 4 and Figure 4.

### 3.3. Factors Contributing to Reduction of Liver Stiffness

A detailed assessment of liver stiffness comparing baseline TE estimates with estimates at SVR12 showed that ≥20% reduction in TE values is more common in patients with non-significant hepatic steatosis. (*p* = 0.05), as well as those who were treatment-naive before starting DAA therapy (*p* = 0.04). More evident decrease in TE values was observed in patients with increased baseline liver enzymes (ALT, *p* = 0.031; AST, *p* = 0.028; GGT, *p* = 0.019). Higher (>14.5 kPa) initial elastography value (*p* = 0.04) and absence of splenomegaly (*p* = 0.031) were also found to be a good predictors of significant TE value decrease. More detailed information is provided in Table A2.

Regression analysis of the data (Table 5) confirms the mentioned results; however, the influence of the degree of histologic hepatic steatosis was marginally insignificant (*p* = 0.053).

### 3.4. Frequency and Risk Factor Characteristics of de Novo HCC Development

During follow-up after the treatment, HCC developed in 4 of 70 patients (5.7%). The previous conventional ultrasound reports of all the HCC patients were consistent with severe hepatic cirrhosis: irregular liver contour and tissue, signs of hepatosteatosis and hepatomegaly. Factors possibly related to the development of HCC included DM2 (*p* = 0.0419), clinically significant elevation in AFP (*p* = 0.0043) and finding of a hypoechoic lesion (*p* = 0.0001). Regression analysis was not used to assess HCC risk factors due to the limited sample size.

## 4. Discussion

The aim of this study was to evaluate patients with advanced liver fibrosis (F3–F4) who require periodic follow-up after DAA treatment. To date, no such studies have been conducted in Lithuania to our knowledge. Our study showed a significant regression of liver stiffness values immediately after achieving SVR. As early as 12 to 24 weeks after the beginning of treatment, patients had an average improvement in the fibrosis stage from the previous F4 to F3 (according to TE values). Comparable results were obtained in other similar studies [9,15,16]. A more pronounced decrease in elastography scores was observed immediately after EoT and less marked at SVR12. This trend is particularly noticeable in patients who had initial elastography values over 14.5 kPa. However, such expressed therapeutic effect may be associated with reduced necroinflammation process in the liver, but not with the regression of fibrosis itself [9]. Baseline FibroScan values in these studies range from 10.2 to 32.5 kPa (25.1 kPa in our study). Such wide distribution of results could be related to the timing of enrollment- in patients with active chronic hepatitis C the TE values are markedly higher [15,16]. However, the reliability of the TE evaluation itself is ambiguous, with only values up to 75 kPa being determined. Thus, the scores of some patients are higher than measurable, but in this case, it is not clear to what extent this affects the results (there were four such patients in our study). It should also be noted that some investigators choose not to include patients with skipped TE examinations in the study sample because they significantly skew the results (this sampling was also used in this study), and for some patients FibroScan examinations fail due to their body habitus or other factors, these were excluded as well.

The HCV genotype 3 has been long associated with a higher risk of developing hepatosteatosis [31]. Viruses of other genotypes also increase the risk of developing steatosis, but indirectly (through association with increased BMI and insulin resistance), whereas, for genotype 3, the degree of HCV viremia is directly associated with steatosis progression [32,33]. The increase in steatosis (as well as a fibrosis) can be measured using elastography. The mentioned association was also observed in this study-patients infected with genotype 3 had significantly higher baseline elastography estimates, but it remains unclear whether due to steatosis or fibrosis.

Associations have been found that may explain the role of liver inflammation (which is also thought to influence changes in the values of the mentioned scales) in the regression of hepatic fibrosis. The significant association between reduced liver stiffness and inflammation (expressed as transaminitis) in this study can be explained by the findings of similar studies on the effect of necroinflammatory response on chronic HCV infection stiffness regression-the greater the inflammation prior to initiating treatment, the better the clinical outcome [34]. Although it remains questionable whether the reduction in inflammatory response or fibrosis regression is measured in the early post-treatment TE examinations, elevated ALT, AST, and GGT are significant in predicting regression of elastography estimates in the early post-treatment period [35,36,37]. However, the use of inflammatory indices to predict a decrease in elastography values is less reliable in patients with decompensated liver cirrhosis (especially with periods of disease exacerbation and elevations in aminotransferases) because the potential for fibrosis regression in such cases is poor [38].

Another significant feature of decompensated liver cirrhosis is splenomegaly. The pathophysiological mechanism that triggers the process is portal hypertension caused by liver fibrosis and inflammation [34,39]. In this study, the reduction in TE values was more often observed in patients without splenomegaly (this is in contrast to other findings which associated higher baseline fibrosis and inflammatory markers with more expressed clinical response). This may be explained by the fact that when portal venous pressure reaches 10–12 mmHg, the progression of portal hypertension largely becomes a spontaneous process independent of hepatic fibrosis [39,40]. Regression of hepatic fibrosis after achieving SVR in these cases becomes less clinically significant. Therefore, US measurement and TE evaluation of spleen are increasingly important in the assessment of particularly advanced liver disease, to predict improvement in patients or to assess regression of portal hypertension after liver transplantation [39,40,41].

The influence of diabetes on the necroinflammatory process of the liver is another important topic. Chronic HCV infection has been associated with an increased risk of developing glucose intolerance, and DM2 has been identified as a factor reducing the potential for fibrosis regression [34,42] and promoting steatogenesis [33,34]. Cases of treatment failure associated with insulin resistance and histological liver damage have also been frequently reported. Some studies have even found an association between circulating insulin concentration and the stage of fibrosis [32,34]. This association may be expressed as a feedback loop in which increased hepatic fibrosis and steatosis lead to increased insulin resistance and vice versa [42]. Even after achieving treatment success and SVR, liver TE values in DM2 patients remain significantly worse [43]. Diagnosing chronic HCV infection in such patients is also complicated as transaminitis in such cases can be misinterpreted as an expression of non-alcoholic steatohepatitis associated with metabolic syndrome. This way, the diagnosis of chronic HCV infection can be delayed to the point of pronounced progression of liver fibrosis. In this study, patients with type 2 diabetes (nearly a quarter of subjects) also had significantly higher TE values before and after treatment, but the effect of DM2 on liver stiffness reduction was not confirmed by regression analysis. Nevertheless, active monitoring of these patients is important in clinical practice, especially in view of the increased risk of developing HCC [23].

Interferon-based treatment leads to fibrosis regression after achieving SVR [6,7,16]. Patients who failed treatment with interferons and were later treated with DAAs are also included in this study. Significant reduction of TE values was observed less frequently in this group than in treatment-naive patients. This is possibly related to a prolonged inflammatory process (due to treatment failure) that has led to decompensated liver cirrhosis in some patients [44]. In addition, interferon therapy has been indicated in patients with advanced hepatic inflammation (to balance the benefits and harms of adverse drug reactions), and fibrosis in such patients often progressed prior to initiating DAA [45]. Thus, the greater reduction in fibrosis in treatment naive patients is probably not a consequence of the direct effects of the drugs themselves, but of the peculiarities of the treatment regimen.

Not only the development of fibrosis but also hepatosteatosis as a result of chronic HCV infection is one of the most widely discussed topics in the field of hepatology in recent years [46]. Methods used in our study could not directly assess the degree of steatosis, as CAP score calculation is unavailable on FibroScan 402; however, a significant association was found between a low degree of steatosis on biopsy (<30%) and an improvement in FS values. It should be noted that DAAs have been associated with the progression of steatosis in some studies even after achieving SVR, with the proportion of steatosis increasing as fibrosis decreases [33]. However, no reliable association was found between the hepatosteatosis and elastography values, and the logistic regression findings were statistically insignificant, requiring more specific methods and a larger sample size for estimating the degree of steatosis in future studies, especially when it is known that liver steatosis influences TE values [47].

One of the main aims of the study was to determine the incidence of hepatocellular carcinoma after DAA treatment. HCC was confirmed in 4 (5.7%) patients (cf. 3–7% in other studies [17,26]). Although a small study sample posed difficulties in statistical analysis, an association was found with HCC risk factors often mentioned in literature—DM2, increased AFP levels, and hypoechoic nodule finding in US examination. Detection of elevated AFP combined with hypoechoic nodule or mass is essential in the diagnosis of HCC with 82.6% sensitivity and 70.4% specificity [48], and confirmation of higher cancer risk in DM2 patients encourages special attention in this group [23].

Our study has several limitations. One of them (in part due to the short time since the introduction of DAAs into clinical practice) is a relatively small sample size. Another limitation is that the TE findings were not corroborated with liver biopsy findings, as histological examinations were not routinely performed after treatment. Since that correlation has not been established, the changes in TE after DAA therapy may not necessarily correlate with findings on biopsy. Some other notable limitations of our study are related to insufficient laboratory test data. We were unable to provide Child-Pugh classification for patients with advanced liver disease due to lack of serum albumin evaluation in a significant number of patients, as at that time, it was not a routine test in our clinic. In this study, we also expected to assess the change in FIB-4 and APRI liver fibrosis scores; however, due to lack of platelet count data at week 12 posttreatment, it was not possible.

## 5. Conclusions

1. Evaluation of elastography values showed a significant decrease in liver stiffness as early as 12 weeks after the end of treatment;
2. Factors linked to more pronounced regression of elastography values were: low percentage of steatosis in liver biopsy, no previous interferon-based treatment, elevated biochemical markers of hepatitis (AST, ALT, and GGT), high baseline elastography measures and absence of splenomegaly;
3. Even after successful DAA treatment, the risk of developing hepatocellular carcinoma seems not to subside completely; therefore, patients should still be monitored closely.
4. The risk groups for non-regressive fibrosis and hepatocellular carcinoma in this study are patients with diabetes mellitus type 2 and advanced liver disease. Correction of risk factors in these patients is particularly important.

## Figures and Tables

**Table 1:** Different direct-acting antiviral drug DAA treatment regimens used in the study.

DAA TREATMENT Scheme	Number of Patients (%)
ombitasvir/paritaprevir/ritonavir/dasabuvir +/− ribavirin	30 (42.9%)
glecaprevir/pibrentasvir	16 (22.9%)
elbasvir/grazoprevir +/− ribavirin *	15 (21.4%)
sofosbuvir/daclatasvir	5 (7.1%)
sofosbuvir/velpatasvir	3 (4.3%)
sofosbuvir/ledipasvir	1 (1.4%)

* ribavirin was included for all patients with confirmed grade F4 fibrosis. The duration of treatment was 12 to 24 weeks, depending on the regimen.

**Table 2:** All patient’s laboratory test data at baseline and at SVR12 ^1^.

Characteristic	Measure
Viremia (baseline), IU/mL	3,380,000 (965,000–6,620,000)
ALT ^2^, U/L	
Baseline	74.8 (47.6–158.3)
SVR12	23.0 (18.3–34.0)
AST ^3^, U/L	
Baseline	85.7 (41.9–144.1)
SVR12	26.0 (21.7–35.5)
GGT ^4^ (baseline), U/L	91.2 (48.2–199.9)
AFP ^5^, kU/L	
Baseline	5.9 (3.8–11.2)
SVR12	4.2 (2.0–6.6)
PTI ^6^ (baseline), %	66 (50–83)
Platelets (baseline), 10^9^/L	131.4 (83.1–179.7)

^1^ SVR12—sustained virologic response after 12 weeks of follow-up; ^2^ ALT—alanine aminotransferase; ^3^ AST—aspartate aminotransferase; ^4^ GGT—γ-glutamyltransferase; ^5^ AFP—alpha-fetoprotein; ^6^ PTI—prothrombin index.

**Table 3:** Changes in liver elastography values.

	Baseline	EoT ^1^	SVR12 ^2^
Median values (kPa)	25.1 (14.5–36.3)	15 (11.4–22)	14.8 (12–27.2)
Median change (kPa)	–	−6.6 (−17.3–−0.9)	−5.6 (−16.1–1.1)
Change, %	–	−26.3%	−22.3%

^1^ EoT—end of treatment; ^2^ SVR12—sustained virologic response after 12 weeks of follow-up.

**Table 4:** Liver and spleen ultrasound findings.

Measure	Result
Baseline liver CC ^1^	1642 (141.0–187.4)
SVR12 ^2^ liver CC	1599 (140.6–179.3)
Baseline spleen length	1200 (110.0–137.5)
SVR12 spleen length	1180 (108.5–135.0)
**Characteristic**	**Number of Patients**
Irregular liver contour	35 (61.4%)
Irregular liver tissue	41 (71.9%)
Signs of hepatosteatosis	25 (43.9%)
Liver focal lesion	8 (14.0%)
Portal lymphadenopathy	5 (8.8%)
Portosystemic collaterals	2 (3.5%)
Ascites	6 (10.5%)
HCC ^3^ (confirmed by CECT ^4^ scan)	4 (5.7%)

^1^ CC—craniocaudal measure; ^2^ SVR12—sustained virologic response after 12 weeks of follow-up; ^3^ HCC—hepatocellular carcinoma; ^4^ CECT—contrast enhanced computed tomography.

**Table 5:** Factors associated with ≥20% decrease in FS ^1^ values. Logistic regression analysis.

Factor	OR ^2^ (95% CI ^3^)	*p* Value
Steatosis in liver bioptate		
≥30% (significant) <30% (non-significant) History of interferon-based treatment	1 3.15 (0.99–10.08)	0.053
Yes	1	
No	3.33 (1.03–10.81)	0.045
ALT ^4^, U/L		
≤40	1	
>40	5.54 (1.12–27.34)	0.036
AST ^5^, U/L		
≤40	1	
>40	6.00 (1.20–30.00)	0.029
GGT ^6^, U/L		
≤36	1	
>36	7.29 (1.31–40.57)	0.023
Baseline FS value, kPa		
≤14.5	1	
>14.5	3.33 (1.03–10.81)	0.045
Splenomegaly		
Yes	1	
No	3.67 (1.10–12.25)	0.035

^1^ FS—FibroScan; ^2^ OR—odds ratio; ^3^ CI—confidence interval; ^4^ ALT—alanine aminotransferase; ^5^ AST—aspartate aminotransferase; ^6^ GGT—gamma-glutamyltransferase.

## Data Availability

The data presented in this study are available on request from the corresponding author. The data are not publicly available due to institutional regulations of Vilnius University Hospital Santaros Klinikos.

## Appendix A

**Table A1:** Patient demographic and clinical data.

Characteristic	*n* (%)
Age, years (mean, ±SD ^1^)	56.3 (44.8–67.8)
Gender:	
Female	28 (40.0%)
Male	42 (60.0%)
BMI ^2^, kg/m^2^	27 (25–32)
<30 kg/m^2^	34 (48.6%)
≥30 kg/m^2^	23 (32.8%)
Unknown	13 (18.6%)
PAH ^3^	
Yes	36 (51.4%)
No	34 (48.6%)
DM2 ^4^	
Yes	17 (24.3%)
No	53 (75.7%)
Alcohol abuse	
Yes	21 (30.0%)
No	49 (70.0%)
Possible route of HCV ^5^ infection	
Blood transfusion	19 (27.4%)
IV drug abuse	19 (27.4%)
Tattoo	15 (21.4%)
Surgical intervention	2 (2.8%)
Unknown	13 (21.0%)
HCV genotype	
1	46 (65.7%)
2	4 (5.7%)
3	18 (25.7%)
HAI ^6^	
4–8	55 (94.8%)
9–12	3 (5.2%)
METAVIR	8 (11.4%)
F3 F4	62 (88.6%)
Degree of steatosis (liver bioptate, median)	20% (7.0–30.0)
History of interferon-based treatment	
No	51 (72.9%)
Yes	19 (27.1%)

^1^ SD—standard deviation; ^2^ BMI—body mass index; ^3^ PAH—primary arterial hypertension; ^4^ DM2—diabetes mellitus type 2; ^5^ HCV—hepatitis C virus; ^6^ HAI—histology activity index.

**Table A2:** Factors without sufficient statistical significance to decrease in elastography values.

Factor	Decreased ≥ 20%, *n =* 38 (%)	Decreased < 20%, *n =* 26 (%)	*p* Value
Age, years 56.3 (44.8–67.8)	–	–	0.289
Gender			
Female	20 (76.9)	16 (42.1)	0.06
Male	6 (23.1)	22 (57.9)	
BMI ^1^, kg/m^2^			
<30	20 (83.3)	24 (63.2)	0.088
≥30	4 (16.7)	14 (36.8)	
PAH ^2^			
No	16 (61.5)	18 (47.4)	0.265
Yes	10 (38.5)	20 (52.6)	
DM2 ^3^			
No	22 (84.6)	30 (78.9)	0.747
Yes	4 (15.4)	8 (21.1)	
Genotype			
1	22 (84.6)	18 (47.4)	0.07
2	2 (7.7)	4 (10.5)	
3	2 (7.7)	16 (42.1)	
METAVIR score			
F3	2 (7.7)	4 (10.5)	0.531
F4	2 (92.3)	34 (89.5)	
Treatment scheme ^4^			
VE	12 (46.2)	10 (26.3)	0.07
MVR	2 (7.7)	16 (42.1)	
ZR	10 (38.5)	6 (15.8)	
SOF	2 (7.7)	6 (15.8)	
Duration of treatment			
12 weeks	24 (92.3)	32 (84.2)	0.456
24 weeks	2 (7.7)	6 (15.8)	
Viremia, IU/mL			
≤600,000	4 (16.7)	2 (5.9)	0.22
>600,000	20 (83.3)	32 (94.1)	
AFP ^5^, kU/L			
≤5.5	14 (63.6)	26 (68.4)	0.705
>5.5	8 (36.4)	12 (31.6)	
Platelets			
≥150 × 109/L	10 (41.7)	8 (21.1)	0.082
<150 × 109/L	14 (58.3)	30 (78.9)	
Signs of steatosis (US ^6^)			
No	20 (76.9)	24 (70.6)	0.582
Yes	6 (23.1)	10 (29.4)	
Hepatomegaly (US)			
No	10 (50.0)	18 (52.9)	0.835
Yes	10 (50.0)	16 (47.1)	

^1^ BMI—body mass index; ^2^ PAH—primary arterial hypertension; ^3^ DM2—diabetes mellitus type 2; ^4^ treatment scheme abbreviations are provided in Table 1; ^5^ AFP—alpha-fetoprotein; ^6^ US—ultrasound. Liver stiffness was evaluated before, after and at SVR12 in 64 of the 70 patients (6 patients had incomplete liver stiffness evaluation).

## References

[1] WHO (2017). Who Global Hepatitis Report.

[2] Rosen H.R. (2011). Chronic Hepatitis C Infection. N. Engl. J. Med..

[3] El-Serag H.B. (2012). Epidemiology of Viral Hepatitis and Hepatocellular Carcinoma. Gastroenterology.

[4] Iacobellis A., Siciliano M., Perri F., Annicchiarico B.E., Leandro G., Caruso N., Accadia L., Bombardieri G., Andriulli A. (2007). Peginterferon alfa- 2b and Ribavirin in patients with hepatitis C virus and decompen-sated cirrhosis: A controlled study. J. Hepatol..

[5] van der Meer A.J., Maan R., Veldt B.J., Feld J.J., Wedemeyer H., Dufour J.F., Lammert F., Duarte-Rojo A., Manns M.P., Zeuzem S. (2016). Improvement of platelets after SVR among patients with chronic HCV infection and advanced hepatic fibrosis. J. Gastroenterol. Hepatol..

[6] George S.L., Bacon B.R., Brunt E.M., Mihindukulasuriya K.L., Hoffmann J., Di Bisceglie A.M. (2008). Clinical, virologic, histologic, and biochemical outcomes after successful HCV therapy: A 5-year follow-up of 150 patients. Hepatology.

[7] Shiratori Y., Imazeki F., Moriyama M., Yano M., Arakawa Y., Yokosuka O., Kuroki T., Nishiguchi S., Sata M., Yamada G. (2000). Histologic Improvement of Fibrosis in Patients with Hepatitis C Who Have Sustained Response to Interferon Therapy. Ann. Intern. Med..

[8] Poynard T., McHutchison J., Manns M., Trepo C., Lindsay K., Goodman Z., Ling M., Albrecht J. (2002). Impact of pegylated interferon alfa-2b and ribavirin on liver fibrosis in patients with chronic hepatitis C. Gastroenterology.

[9] Knop V., Hoppe D., Welzel T., Vermehren J., Herrmann E., Friedrich-Rust M., Sarrazin C., Zeuzem S., Welker M.-W. (2016). Regression of fibrosis and portal hypertension in HCV-associated cirrhosis and sustained virologic response after interferon-free antiviral therapy. J. Viral Hepat..

[10] Scaglione V., Mazzitelli M., Costa C., Pisani V., Greco G., Serapide F., Lionello R., La Gamba V., Marascio N., Trecarichi E.M. (2020). Virological and Clinical Outcome of DAA Containing Regimens in a Cohort of Pa-tients in Calabria Region (Southern Italy). Medicina.

[11] Alimohammadi A., Holeksa J., Thiam A., Truong D., Conway B. (2018). Real-world Efficacy of Direct-Acting Antiviral Therapy for HCV Infection Affecting People Who Inject Drugs Delivered in a Multidisciplinary Setting. Open Forum Infectious Diseases.

[12] Afdhal N., Everson G.T., Calleja J.L., McCaughan G., Bosch J., Denning J., Brainard D.M., McHutchison J.G., Brandt-Sarif T., An D. (2015). Effect of long term viral suppression with Sofosbuvir and Ribavirin on hepatic venous pressure gradient in HCV- infected patients with cirrhosis and portal hypertension. J. Hepatol..

[13] Actis G.C., Olivero A., Lagget M., Pellicano R., Smedile A., Rizzetto M. (2007). The practice of percutaneous liver biopsy in a gastrohepatology day hospital: A retro-spective study on 835 biopsies. Dig. Dis. Sci..

[14] Rockey D.C., Caldwell S.H., Goodman Z.D., Nelson R.C., Smith A.D. (2008). Liver biopsy. Hepatology.

[15] Bachofner J.A., Valli P.V., Kröger A., Bergamin I., Künzler P., Baserga A., Braun D., Seifert B., Moncsek A., Fehr J. (2017). Direct antiviral agent treatment of chronic hepatitis C results in rapid regression of tran- sient elastography and fibrosis markers fibrosis- 4 score and aspartate aminotransferase- platelet ratio index. Liver Int..

[16] Tada T., Kumada T., Toyoda H., Mizuno K., Sone Y., Kataoka S., Hashinokuchi S. (2017). Improvement of liver stiffness in patients with hepatitis C virus infection who received di-rect- acting antiviral therapy and achieved sustained virological response. J. Gastroenterol. Hepatol..

[17] Roche B., Coilly A., Duclos-Vallee J.C., Samuel D. (2018). The impact of treatment of hepatitis C with DAAs on the occurrence of HCC. Liver Int..

[18] Innes H.A., McDonald S.A., Dillon J.F., Allen S., Hayes P.C., Goldberg D., Mills P.R., Barclay S.T., Wilks D., Valerio H. (2015). Toward a more complete understanding of the association between a hepatitis C sus-tained viral response and cause-specific outcomes. Hepatology.

[19] van der Meer A.J., Veldt B.J., Feld J.J., Wedemeyer H., Dufour J.F., Lammert F., Duarte-Rojo A., Heathcote E.J., Manns M.P., Kuske L. (2012). Association between sustained virological response and all- cause mortality among patients with chronic hepatitis C and advanced hepatic fibrosis. JAMA.

[20] Bruno S., Shiffman M.L., Roberts S.K., Gane E.J., Messinger D., Hadziyannis S.J., Marcellin P. (2010). Efficacy and safety of peginterferon alfa-2a (40KD) plus ribavirin in hepatitis C pa-tients with advanced fibrosis and cirrhosis. Hepatology.

[21] Simmons B., Saleem J., Heath K., Cooke G.S., Hill A. (2015). Long-Term Treatment Outcomes of Patients Infected With Hepatitis C Virus: A Systematic Review and Meta-analysis of the Survival Benefit of Achieving a Sustained Virological Response. Clin. Infect. Dis..

[22] Nahon P., Bourcier V., Layese R., Audureau E., Cagnot C., Marcellin P., Guyader D., Fontaine H., Larrey D., De Lédinghen V. (2017). Eradication of Hepatitis C Virus Infection in Patients With Cirrhosis Reduces Risk of Liver and Non-Liver Complications. Gastroenterology.

[23] El-Serag H.B., Kanwal F., Richardson P., Kramer J. (2016). Risk of hepatocellular carcinoma after sustained virological response in Veterans with hepatitis C virus infection. Hepatology.

[24] Reig M., Mariño Z., Perelló C., Iñarrairaegui M., Ribeiro A., Lens S., Díaz A., Vilana R., Darnell A., Varela M. (2016). Unexpected high rate of early tumor recurrence in patients with HCV-related HCC undergo-ing interferon-free therapy. J. Hepatol..

[25] Guarino M., Sessa A., Cossiga V., Morando F., Caporaso N., Morisco F. (2018). Direct-acting antivirals and hepatocellular carcinoma in chronic hepatitis C: A few lights and many shadows. World J. Gastroenterol..

[26] Conti F., Buonfiglioli F., Scuteri A., Crespi C., Bolondi L., Caraceni P., Foschi F.G., Lenzi M., Mazzella G., Verucchi G. (2016). Early occurrence and recurrence of hepatocellular carcinoma in HCV-related cirrhosis treated with direct-acting antivirals. J. Hepatol..

[27] Waziry R., Hajarizadeh B., Grebely J., Amin J., Law M., Danta M., George J., Dore G.J. (2017). Hepatocellular carcinoma risk following direct-acing antiviral HCV therapy: A sys-tem- atic review, meta-analyses, and meta-regression. J. Hepatol..

[28] Ioannou G.N., Green K., Berry K. (2017). HCV eradication induced by direct-acting antiviral agents reduces the risk of hepatocellular carcinoma. J. Hepatol..

[29] Kanwal F., Kramer J., Asch S.M., Chayanupatkul M., Cao Y., El-Serag H.B. (2017). Risk of Hepatocellular Cancer in HCV Patients Treated With Direct-Acting Antiviral Agents. Gastroenterology.

[30] Dolmazashvili E., Abutidze A., Chkhartishvili N., Karchava M., Sharvadze L., Tsertsvadze T. (2017). Regression of liver fibrosis over a 24-week period after completing di-rect- acting antiviral therapy in patients with chronic hepatitis C receiving care within the national hepatitis C elimination program in Georgia: Results of hepatology clinic HEPA experience. Eur. J. Gastroenterol. Hepatol..

[31] Rubbia-Brandt L., Quadri R., Abid K., Giostra E., Malé P.J., Mentha G., Spahr L., Zarski J.P., Borisch B., Hadengue A. (2000). Hepatocyte steatosis is a cytopathic effect of hepatitis C virus genotype 3. J. Hepatol..

[32] Fartoux L., Poujol-Robert A., Guechot J., Wendum D., Poupon R., Serfaty L. (2005). Insulin resistance is a cause of steatosis and fibrosis progression in chronic hepa-titis C. Gut.

[33] Rout G., Nayak B., Patel A.H., Gunjan D., Singh V., Kedia S. (2019). Therapy with Oral Directly Acting Agents in Hepatitis C Infection Is Associated with Reduc-tion in Fibrosis and Increase in Hepatic Steatosis on Transient Elastography. J. Clin. Exp. Hepatol..

[34] Persico M., Rosato V., Aglitti A., Precone D., Corrado M., De Luna A., Morisco F., Camera S., Federico A., Dallio M. (2018). Sustained virological response by direct antiviral agents in HCV leads to an early and signif- icant improvement of liver fibrosis. Antivir Ther..

[35] Berzigotti A. (2017). Non-invasive evaluation of portal hypertension using ultrasound elastography. J. Hepatol..

[36] Coco B., Oliveri F., Maina A.M., Ciccorossi P., Sacco R., Colombatto P., Bonino F., Brunetto M.R. (2007). Transient elastography: A new surrogate marker of liver fibrosis influenced by major changes of transaminases. J. Viral Hepat..

[37] Sagir A., Erhardt A., Schmitt M., Häussinger D. (2007). Transient elastography is unreliable for detection of cirrhosis in patients with acute liver damage. Hepatology.

[38] Bruno S., Stroffolini T., Colombo M., Bollani S., Benvegnu L., Mazzella G., Ascione A., Santantonio T., Piccinino F., Andreone P. (2007). Sustained virological response to interferon-alpha is associated with improved out-come in HCV-related cirrhosis: A retrospective study. Hepatology.

[39] Giunta M., Conte D., Fraquelli M. (2016). Role of spleen elastography in patients with chronic liver diseases. World J. Gastroenterol..

[40] Castera L., Pinzani M., Bosch J. (2012). Non invasive evaluation of portal hypertension using transient elastography. J. Hepatol..

[41] Elshaarawy O., Mueller J., Guha I.N., Chalmers J., Harris R., Krag A., Madsen B.S., Stefanescu H., Farcau O., Ardelean A. (2019). Spleen stiffness to liver stiffness ratio significantly differs between ALD and HCV and predicts disease-specific complications. JHEP Rep..

[42] Hammerstad S.S., Grock S.F., Lee H.J., Hasham A., Sundaram N.K., Tomer Y. (2015). Diabetes and Hepatitis C: A Two-Way Association. Front. Endocrinol..

[43] Serfaty L. (2016). Follow-up of patients with chronic hepatitis C and a sustained viral response. Liver Int..

[44] Foster G.R., Irving W.L., Cheung M.C., Walker A.J., Hudson B.E., Verma S., McLauchlan J., Mutimer D.J., Brown A., Gelson W.T. (2016). Impact of direct acting antiviral therapy in patients with chronic hepatitis C and de-com- pensated cirrhosis. J. Hepatol..

[45] Carmona I., Cordero P., Ampuero J., Rojas A., Romero-Gómez M. (2016). Role of assessing liver fibrosis in management of chronic hepatitis C virus infection. Clin. Microbiol. Infect..

[46] Rosso C., Caviglia G.P., Younes R., Ribaldone D.G., Fagoonee S., Pellicano R., Bugianesi E. (2020). Molecular mechanisms of hepatic fibrosis in chronic liver diseases. Minerva Biotecnol..

[47] Chen C.-J., Tsay P.-K., Huang S.-F., Tsui P.-H., Yu W.-T., Hsu T.-H., Tai J., Tai D.-I. (2019). Effects of Hepatic Steatosis on Non- In-vasive Liver Fibrosis Measurements between Hepatitis B and Other Etiologies. Appl. Sci..

[48] Chan S.L., Mo F., Johnson P.J., Siu D.Y.W., Chan M.H.M., Lau W.Y., Lai P.B.S., Lam C.W.K., Yeo W., Yu S.C.H. (2014). Performance of serum α-fetoprotein levels in the diagnosis of hepatocellular carcinoma in patients with a hepatic mass. HPB.

